# Grounding the Restorative Effect of the Environment in Tertiary Qualities: An Integration of Embodied and Phenomenological Perspectives

**DOI:** 10.3390/jintelligence11110208

**Published:** 2023-10-31

**Authors:** Federica Biassoni, Michela Gandola, Martina Gnerre

**Affiliations:** 1 Traffic Psychology Research Unit, Department of Psychology, Università Cattolica del Sacro Cuore, 20123 Milano, Italymartina.gnerre@unicatt.it (M.G.); 2 Research Center in Communication Psychology, Università Cattolica del Sacro Cuore, 20123 Milan, Italy

**Keywords:** restorativeness, embodied cognition, phenomenological perspective, experimental phenomenology, tertiary qualities

## Abstract

This paper proposes an integration of embodied and phenomenological perspectives to understand the restorative capacity of natural environments. It emphasizes the role of embodied simulation mechanisms in evoking positive affects and cognitive functioning. Perceptual symbols play a crucial role in generating the restorative potential in environments, highlighting the significance of the encounter between the embodied individual and the environment. This study reviews Stress Reduction Theory (SRT) and Attention Restoration Theory (ART), finding commonalities in perceptual fluency and connectedness to nature. It also explores a potential model based on physiognomic perception, where the environment’s pervasive qualities elicit an affective response. Restorativeness arises from a direct encounter between the environment’s phenomenal structure and the embodied perceptual processes of individuals. Overall, this integrative approach sheds light on the intrinsic affective value of environmental elements and their influence on human well-being.

## 1. Introduction 

In this essay, the authors define for the first time the concept of ‘Phenomenological restorativeness’ (PR). PR combines elements of phenomenology and environmental psychology to understand how the perception of natural environments may contribute to feelings of restoration and well-being. It concentrates on the immediate and often subconscious affective responses individuals experience when they interact with natural environments.

The central idea behind PR is that these innate, immediate affective responses to specific environmental qualities can play a crucial role in reducing stress, enhancing attention, and promoting well-being. It emphasizes the importance of understanding how these perceptual characteristics of natural environments contribute to their restorative effects and how they interact with the embodied, sensory–motor processes of individuals.

PR explicitly emphasizes the inherent perceptual and aesthetic and affective qualities of environments that evoke restorative effects, and acknowledges the affective nature of primary responses to the environment, bridging the gap between immediate perceptual experiences and cognitive processes.

The goal of this paper is to integrate phenomenological restorativeness with the Biophilia Hypothesis (Wilson 1986) and the explanatory restorativeness theories, notably The Stress Recovery Theory (SRT) by (Ulrich 1983; Ulrich et al. 1991) and the Attention Restoration Theory (ART) by Kaplan and Kaplan (1989), Kaplan (1995). This integration will be explored within the framework of embodied perception and individual–environment interactions.

It investigates how tertiary-expressive qualities of the environment, which cause instantaneous affective reactions, can provide healing benefits. This essay emphasizes that the restorative capacity of the natural environment emerges from the encounter between its phenomenal structures and the embodied perceptual and affective system. By doing so, it seeks to provide epistemic support to the embodied nature of the environment’s restorative qualities.

The embodied cognition perspective definitively nourishes our understanding and confirms the embodied nature of the restorative qualities of the environment. On the one hand, this perspective contributes to overcoming a regenerative view of the environment that is somehow still Cartesian, and on the other hand, it substantiates the phenomenological tradition that anticipated the embodied paradigm of knowledge.

### Environmental Stress and Restorativeness in Natural and Urban Environments

Environmental stress, from a psychological perspective, encompasses the multitude of specific features causing pressures and disturbances that can impact cognitive, emotional, and physiological resources (Evans 1984). Traditionally, some of these features have been identified in the noise, crowding, and pollution in urban settings (Meloni et al. 2019), but research has also highlighted that some natural environments have characteristics that make them undesirable (Andrews and Gatersleben 2010). These stress-inducing factors have significant implications for well-being, as chronic stress has been associated with the development of various non-communicable diseases, including cardiovascular disease, metabolic disorders, immune dysfunction, cancer, and psychiatric disorders, as well as a decrease in overall quality of life (Peña-Oyarzun et al. 2018). Conversely, exposure to restorative environments, such as serene forests, peaceful beaches, vibrant gardens, or well-designed urban green spaces, can provide individuals with a sense of relaxation, rejuvenation, and revitalization (Townsend et al. 2018).

Moreover, it is important to consider that nature can be seen as a continuum, ranging from fully built to untouched natural areas. This perspective blurs the traditional boundary between what is considered “natural” and “urban”. This perspective challenges the dichotomy between these two categories and highlights the idea that there is a spectrum or continuum of environments, each with varying degrees of human influence. Less managed natural areas can offer opportunities for adventure and exploration but can also promote a sense of risk and vulnerability. Therefore, it is important to recognize the varying restorative potentials of different types of environments. It is important to consider that not all individuals find restoration in pristine natural settings; built and mixed urban environments can provide a sense of comfort, familiarity, and safety, that fosters a sense of belonging (Patuano 2020).

Research has predominantly emphasized the restorative potential of entirely natural settings while allocating less attention to urban or mixed environments (Ulrich et al. 1991; Kaplan 1992; Karmanov and Hamel 2009). 

In fact, many studies have shown that exposure to natural environments can have direct and indirect effects on stress recovery and mental fatigue restoration, fostering measures to promote healthy lifestyles and achieve physical, emotional, and attentional balance (Lee et al. 2021; Berto 2014; Bowler et al. 2010; Calogiuri and Chroni 2014; Corazon et al. 2019). When a natural setting encourages a transition to more uplifting emotional states, beneficial adjustments in physiological activity levels, and enhancements in behavior and cognitive performance, it is referred to as a “restorative environment” (Ulrich et al. 1991; Kaplan 1992). Wadeson et al. (1963) showed that exposure to natural environments resulted in a reduction of cortisol levels, and recent research shows that exposure to natural stimuli in any form can lead to a reduction in symptoms related to psycho-physiological stress and irritability (Lee et al. 2021). Experiencing nature can enhance cognitive functioning and reduce cognitive overload, facilitating behaviors that foster inhibition, patience, and endurance, which are, in turn, essential for completing challenging tasks (Berto 2014; Kaplan and Kaplan 1989). This can lead to numerous positive outcomes, including improved performance and enhanced planning abilities (Berman et al. 2008; Lee et al. 2021). Being in nature can also have a positive effect on social behavior (Bratman et al. 2012), emotional regulation, and pro-environmental behavior (Panno et al. 2020).

Recently, there has been a growing interest in the restorative potential of urban and mixed environments as well, highlighting the concept of urban restorativeness and the significance of designing cities that promote relaxation and well-being among their residents (Patuano 2020). Although numerous studies have proved that natural environments are more restorative than human-built environments (for a meta-analysis see Menardo et al. 2021), other studies have explored the restorative potential of built and mixed urban environments (Troffa and Fornara 2011; Karmanov and Hamel 2009). Any setting that has restorative properties may be a restorative one (Kaplan 1995). In fact, an attractive and well-designed urban setting may have the same calming and uplifting effects as a beautiful natural setting. It appears that the perceived restorative effects of urban environments cannot be solely attributed to the presence of water and green spaces, but also to other factors such as the design of the urban environment, the intricate spatial layout of the area, and the presence of landmarks (Karmanov and Hamel 2009).

## 2. Two Theoretical Perspectives on Environmental Restorativeness

The relationship between humans and nature has been a subject of interest and study for centuries. Throughout history, we have depended on nature for our basic needs, but its importance to our overall well-being goes beyond mere survival.

Different theories have been developed: for example, according to Orians’ Savanna Hypothesis (Orians 1980, 1986), humans have an innate preference for landscapes resembling the ancestral savanna environment in which our species evolved (Bennett 2019). In addition to the Savanna Hypothesis, there are two complementary frameworks that shed light on the profound benefits of restorative environments: The Stress Recovery Theory (SRT), developed by (Ulrich 1983; Ulrich et al. 1991), and the Attention Restoration Theory (ART), proposed by Kaplan and Kaplan (1989), Kaplan (1995). Both theories are based on the Biophilia Hypothesis (Kellert and Wilson 1993; Wilson 1986), which suggests that humans have an innate tendency to respond positively to natural environments due to their evolutionary history.

SRT is a psycho-evolutionary hypothesis that indicates that physiological stress is the primary motivation for individuals to seek out natural environments. According to this theory, when people are exposed to natural environments, they experience a positive emotional response and a reduction in stress, leading to restorative outcomes. The idea is that this reaction was beneficial for our ancient human ancestors, as it helped them in their search for necessary resources (such as food, water, shelter) and mitigated the negative impact of stressors in their environment (Ulrich 1993). The SRT (Ulrich et al. 1991; Ulrich 1993) posits that humans are physically and mentally suited to natural environments due to spending a significant amount of time evolving in natural surroundings (Ulrich 1993). According to this theory, exposure to natural stimuli has an immediate impact on our emotions, triggering a response in the parasympathetic nervous system (Clatworthy et al. 2013). Viewing natural environments reduces blood pressure and heart rate (Laumann et al. 2003), and decreases perspiration and muscular tension (Ulrich et al. 1991). The SRT posits that humans are physically and mentally suited to natural environments for evolutionary reasons (Ulrich 1993), as it helped them in their search for necessary resources (such as food, water, shelter) and mitigated the negative impact of stressors in their environment (Ulrich 1993). Ulrich’s (1983) theory focuses on the influence of nature on both emotional and physiological functioning (Clatworthy et al. 2013). From this viewpoint, our attention is influenced by our rapid, unconscious emotional reactions, rather than more deliberate cognitive processes. On the contrary, natural environments’ features like wide spaces, low density, and open, unobstructed views, would produce the opposite effect of the stimulating patterns of parasympathetic arousal, and subjectively positive feelings. Such features were originally connected to favorable conditions for settlement and this primal positive value persists, so that our immediate and unconscious emotional responses can influence our attention, physiology, and behavior, influencing the rapid attenuation of stress responses and the quick recharge of physical energy, which in turn had significant evolutionary advantages. Elements (such as water and vegetation) which were originally fundamental for survival, immediately suggest natural environments are safe. Aesthetically pleasing built settings containing water and prominent vegetation might have a restorative influence similar to natural scenes (Ulrich 1993).

This theory relates environmental stress to an increase in arousal and in negative emotion, and proposes that recovery from excessive arousal or stress should occur more rapidly in settings with low levels of arousal-increasing properties (Ulrich et al. 1991). From this perspective, the urban environment, can, in certain situations, impede our capacity for relaxation due to its complexity and stimulating characteristics that induce increased alertness. The opposite is also true: an intricate spatial layout may induce a sense of mystery and suggest an opportunity for exploration, unsurprisingly, the interestingness ratings of the urban environment significantly exceeded those of the natural environment (Karmanov and Hamel 2009). Moreover, individuals suffering from excessively low arousal or chronic boredom might benefit from being exposed to lively and stimulating urban views (Patuano 2020). Within the realm of SRT, those afflicted with excessively low arousal or chronic boredom may discover relief and an enhancement in their psychological state through exposure to lively and stimulating urban views (in line with the Yerkes–Dodson Law). This synergy with the Yerkes–Dodson Law implies that individuals with low arousal levels, as seen in the case of boredom, can benefit from the introduction of a moderate level of stimulation, represented in this case by urban views. This, in turn, can help them approach the optimal arousal level, leading to an enhancement in their well-being.

ART, on the other hand, is a psycho-functional theory that focuses on mental fatigue and uses an information-processing approach. This theory posits that natural environments are characterized by features such as fascination, extent, and coherence, which capture our attention without requiring effortful attention, and thus allow for restorative experiences. The Kaplans claim that there are two different types of attention people use in everyday life: one is directed attention, employed in many tasks including driving, working, and looking for their keys, the other one is effortless attention, also known as “soft fascination,” which is a less directed type of attention in which our mind is free to “rest and wander freely” (Kaplan and Kaplan 1989; Kaplan 1995; Kaplan and Berman 2010). When directed attention is employed, the greatest threat to maintaining a given focus is competition from other stimuli that can cause a shift in focus. This is because one maintains focus on a specific task by inhibiting all potential distractions represented by concurring stimuli. The directed attention’s capacity is limited, requires a great deal of effort, and quickly is exhausted. Hence, directed attention fatigue occurs when a particular part of the brain’s global inhibitory system is overworked due to the suppression of increasing numbers of stimuli. The quality of directed attention degrades over a specific period of time or after a particular volume of data, and a great deal of focus and effort inevitably leads to mental fatigue. The mental fatigue state increases the probability that an individual experiences the stress response due to cognitive overload, and the concomitant reduction of the cognitive resources necessary to address daily requests. Mental fatigue manifests itself in negative emotions, irritability, impulsiveness, impatience, reduced tolerance for frustration, insensitivity to interpersonal cues, decreased altruistic behaviors, reduced performance, increased likelihood of taking risks, and, generally speaking, in reduced competence and/or decreased effectiveness in functioning. In practice, the inability to renew the attentional capacity aggravates the mental fatigue state and can also adversely impact mood, work performance, and interpersonal relationships.

When experiencing mental fatigue, individuals tend to show a stronger inclination and preference towards natural environments compared to urban ones (Hartig and Staats 2005). This is because natural surroundings are particularly suited to engaging our involuntary attention, whereas built environments can be highly attention-capturing, necessitating a conscious effort to overcome distractions and, therefore, a large deployment of directed attention. Some studies have provided support for the restoration potential of environments that elicit fascination. For instance, Berman et al. (2008) conducted a study where participants performed a cognitive task, followed by a walk either in a natural setting or an urban setting. They found that individuals who walked in nature showed improved performance in subsequent cognitive tasks compared to those who walked in urban environments. The researchers attributed this restoration effect to the attentional benefits derived from the fascination of the natural environment. Similarly, Joye and van den Berg (2011) explored the impact of natural environments on mental restoration with evolutionary assumptions. They showed that individuals exposed to natural environments experienced a significant decrease in self-reported mental fatigue compared to those exposed to urban environments, because restoration is an ancient adaptive response. The researchers attributed this finding to the restorative qualities of natural environments that fostered fascination and effortless attention. 

In sum, the two theories are two different ways to explain related but distinct phenomena. SRT primarily focuses on how natural environments can reduce stress and promote relaxation, while ART focuses on how exposure to nature can restore attention and cognitive function. While they both highlight the positive effects of nature, they address different aspects of human well-being and functioning. Despite their differences, the two theories complement each other and can be used in combination to design restorative environments. 

## 3. An Embodied Cognition Perspective on Restorativeness

The embodied paradigm was prefigured by Husserl, who posited that consciousness is always directed towards something and inherently “intends” phenomena. This perspective was further developed by Merleau-Ponty, who seems to be the philosopher most deeply engaged in shaping the embodied paradigm. The French philosopher ascribes primary importance to perception (and the body) in the cognitive process, effectively giving precedence to experience. This is based on the premise that consciousness is fundamentally rooted in perception (Merleau-Ponty 1945).

In accordance with Merleau-Ponty’s philosophy, the body serves as the intermediary through which we form connections and engage with the world (Thompson 2007). Rediscovering the body entails a rediscovery of both the perceived world and the entire subject–object relation. That is, once the body is subtracted from the objective world, not only is the perceiving subject revealed, but also the perceived world. Merleau-Ponty’s works on corporeality, on the historical and intersubjective grounding of the individual in the lived (experienced) world, anticipated the future evidence provided by neuroscience. Neuroscience itself, in turn, through simulated neural dynamics, seems to support this phenomenological perspective (Gallese 2009). The Gestalt theorists (Köhler 1929) proposed the concept of isomorphism, suggesting that there is a corresponding neurobiological form and dynamic (embodied) for every experiential form and dynamic. In the Gestalt perspective, perception is not merely the sum of individual sensory elements; it transcends them and is also an immediate and automatic process (Koffka 1935). Interestingly, the idea of perception as related to automatic processes regulated by experiential scripts, as well as the concept of isomorphism, evoke the concept of embodied simulation. In fact, both concepts emphasize the simultaneous existence and alignment of neurobiological, sensorimotor, and phenomenological mechanisms, as claimed by Mungan: “This is why it is so sad and quite baffling that the Varela team had not read the Gestaltists despite the fact that they intensively read Merleau-Ponty. So, what is missing in the Varela et al. conceptualization? I think what is missing is information about the specific dynamics that bring about perception … for instance, figure-ground perception and perceptual grouping…” (Mungan 2023, p.13).

Embodied cognition, by reinstating the significance of the body in mental processes and attributing mental qualities to the body, suggests a relationship that extends beyond the mind–body connection. It also encompasses the relationship between humans and their environment, where the brain is not the sole, exclusive resource, neither in terms of cognition nor relational aspects. This term does not denote a single, fixed theory but rather represents a broad field of interdisciplinary research. Despite their internal differences, it is clear that cognitive processes represent the common foundation, include broader body structures and interactive processes with the environment, and are not confined to operations present in the cognitive system (Lakoff and Johnson 1999; Noë 2004; Chemero 2009). Within this psycho-evolutionary matrix, dispositional-experiential mechanisms (cognitive, affective, motivational, etc.), universal mechanisms (simulative dynamics, survival tendencies, etc.), and historical-cultural-symbolic determinants come into play. The understanding of embodied cognition as presented here revolves around the concept of embodiment, which is seen as an extended relational condition involving the body, mind, and environment. This perspective is in line with the ideas of Clark and Chalmers (1998), who view embodiment as ecologically situated, evolutionary, and autopoietic (Varela et al. 2017). With this foundation established, we can now delve into the relational aspects that define the “embodied self-world bond” (Varela et al. 2017), which forms the fundamental concept of phenomenological restorativeness.

### 3.1. Simulation as the Foundation for the Individual-Environment Relationship

The first relational aspect concerns the embodied nature of hedonic experience, which involves a connection between embodied simulation and aesthetic perception (Gallese and Gattara 2021). Here, aesthetic perception relates to the aspect of knowledge that engages our senses. This relationship involves the neural mechanisms responsible for simulative processes that display an empathic capacity extending beyond just social interactions. Mirror mechanisms demonstrate an ability to transform perceptual experiences into personal knowledge, which encompasses not only procedural elements but also intentional, emotional, and sensory components. This is in support of the notion that this simulative model explains intersubjectivity, not exhaustively, but in many of its bodily qualities. The schema “as if” seems to characterize all forms of intentional relationships (Gallese 2011). In this framework, the perceiving individual possesses the ability to understand what is observed, heard, or imagined, because they already have experiential knowledge of it. This implies a relationality, not solely biologically grounded, between the external world as perceived and the internal experiential heritage.

The role of embodied simulation becomes even more significant when we consider the emotional and affective aspects of our experiences in the environment. An important perspective in this regard comes from the work of Damasio, who illustrates how our entire subjective experience is rooted in our embodiment. According to Damasio, there is no such thing as completely neutral perception because, in neural terms, it tends to be predisposed to elicit an emotional–affective response even before cognitive interpretation occurs. Damasio’s somatic marker hypothesis suggests that the connection between the self and the world initially involves an emotional and bodily relational response, with cognition coming into play later. In our encounters with the world, these emotional and somatic responses, create a mnemonic deposit that informs and influences our subsequent interactions with the self and the world (Damasio 2007; Gallese 2011). When the perceived environment interacts with an individual’s embodied and experiential self through the simulative mechanism, it has the capacity to evoke patterns of past experiences stored within neurobiological, perceptual, cognitive–emotional, and cultural frameworks. This evocation of experiential patterns can trigger positive emotional responses, consequently enhancing the individual’s well-being. This process relies on the simultaneous interplay of two key factors. On one hand, there is the emotional–affective dimension inherent in every form of sensorimotor interaction with the world, as explained by Damasio (2012). On the other hand, there is the sensorimotor relationship itself, which is, in turn, characterized by simulation mechanisms, as described by Freedberg and Gallese (2007). Consequently, perceptual experiences not only qualify as affective–hedonic encounters but also as bodily experiences. This viewpoint asserts a perception-grounded origin of knowledge, which finds expression in a reimagined understanding of the environment. The environment becomes a source of experiential generation for cognitive and emotional–affective senses and meanings, as well as perceptual and motor references (Lingiardi 2017, p. 8).

### 3.2. The Environment as an “Embodied Place”

The second relational aspect concerns the concept of an environment evolving into an “embodied place” with “restorative potential.” This environment, when encountered through perceptual experiences, has the capacity to not only foster personal flourishing, as described by Seligman (2012), but also to facilitate a kind of “environmental flourishing.” In these terms, environments embody qualities capable of eliciting experiential potentials that have been previously lived or imagined and “incorporated” with an ontogenetic and cognitive–emotional connection (Orians and Heerwagen 1992; Orians 1980; Kellert and Wilson 1993). Psychological research suggests that attachment bonds represent the relational patterns that recur and shape our subsequent relationships. Therefore, it is reasonable to hypothesize (Casakin and Bernardo 2011; Proshanski 1983; Gallino 2007) that the attachment bond with the environment can, in a perceptual encounter, reactivate similar past experiences with a direct and automatic intersubjectivity that links historical experiences to current ones. In this embodied perspective, the environmental element is not just a stimulus with evocative potential, but also a stimulus with affective–cognitive content, linked to those experiences already lived by the subject.

Similarly, on a more universal scale, the environment, such as a historical village or a river, can encapsulate not only an individual’s experiences but also the cultural and symbolic experiences of an entire species. In this case, the environmental element possesses qualities imbued with an evocative power connected to universal cognitive–affective affiliations, as suggested by Mallgrave (2013). We are, fundamentally, embodied beings whose body, mind, environment, and culture are interconnected in various ways. Drawing from Barsalou’s work (1999), it can be hypothesized that the embodied environment includes factors—both natural and symbolic, aesthetic and geometric (Pallasmaa 2014)—that stimulate perceptual and imaginative experiences. Consequently, the observer intentionally and phenomenologically engages with the world. The underlying idea is that the relationship with the environment emerges from the degree of alignment between the structural relationships expressed by the environment and the concurrent, responsive, neurobiological modeling that arises from the activation of sensorimotor patterns elicited by the environment itself. As we engage with our environment through our senses, we not only simulate its physical forms and colors with our bodies, but we also tend to internalize and embody the deeper meanings embedded in the world. Thus, when visiting a place like the Palace of Versailles, the embodied experience can emerge from the qualities inherent in the environment, stirring imaginative potentials within the individual, as if they were transported to a dance in the Hall of Mirrors. The deeply ingrained and embodied images within the tapestry of the world carry an evocative symbolic power capable of conveying archetypal meanings, which become embodied within the individual (Hillman and Truppi 2004).

Barsalou (1999) introduces the term “perceptual symbols” to describe the sensorimotor and affective representations through which we engage with, comprehend, and envision objects within our environment. According to Barsalou, cognition is intricately linked to perception, making it inherently perceptual. In this framework, concepts act as “simulators” because they simulate various experiences we might have with events or objects in the brain (and in the body). Consequently, the meaning of an object extends beyond its abstract concept, encompassing all the real or imagined experiences associated with it, including personal symbolization. This process of assigning meaning and the resulting restorative power of the environment are elucidated through these simulative, multimodal, and enactive processes.

Embodied relationality can be summarized by acknowledging that both humans and the environment carry within them embodied experiences, which, during their relational encounters, transform into present subjective experiences. The emergence of the restorative power and the subjective experience of well-being hinges on the degree of alignment between the embodied qualities of the environment and the embodied subjectivity of the individual. Certain environmental qualities possess restorative properties because they foster a relationship with facets of ourselves that become nourished, subsequently contributing to our overall well-being. Indeed, the restorative effects of the environment are fundamentally linked to the harmonious interaction between the environmental context and our embodied selves.

## 4. Restorativeness as a Tertiary-Expressive Quality

The perception of environmental elements and natural landscapes has been a subject of interest in Gestalt psychology and later in experimental phenomenology as well (Bozzi 1989; 2002, translated in Bianchi and Davies 2019, pp. 11–45; Michotte 1946). Interestingly, the perception of natural elements and landscapes has been explored with a clear reference to their ability to evoke affective reactions in the observer, who encounters their characteristics through perception, known as “tertiary qualities” (Bozzi 1998; 1990, translated in Bianchi and Davies 2019, pp. 345–68; Verstegen and Fossaluzza 2019). These are a set of qualities of the object immediately perceived as intrinsic value qualities, regardless of whether they are positive or negative. Kurt Lewin, who was a precursor to some key concepts in environmental psychology, had already referred to certain perceptive properties of the phenomenal field as “inviting or repulsive characteristics,” describing the affective nature produced by the encounter between these properties and the perceptive functioning of the human being.

The term “tertiary qualities” refers to pre-categorical qualities of immediate experience, not reducible to the physical dimension of the object or the subject’s experience. We are talking about phenomenal qualities. As Bozzi states, they are the characteristics of an object that “attract” certain adjectives because their perception leads to specific affective reactions in the perceiver.

*If black is gloomy, red is vibrant. The shade of a large green tree is relaxing and soothing. A diminished seventh chord is tense and curling. A slow and ascending gesture is hieratic. We are not simply attaching stereotyped adjectives to simple facts; in those facts, there are characteristics that inherently attract those adjectives, and these characteristics are not verbal or associative in nature but perceptual ingredients present within the facts themselves. These ingredients emerge from the facts with immediate evidence […]. “Everything says what it is,” wrote K. Koffka, “… a fruit says ‘eat me’; water says ‘drink me’; thunder says ‘be afraid of me’; and a woman says ‘love me.’” (Koffka 1935).*

(Bozzi 1998, p. 100).

These properties manifest as qualities of the objects, making them appear intrinsically imbued with a dimension of value and meaning. Encountering and grasping these properties through our perception systems inevitably means being affected by their meaningful dimension. The immediate and direct experience of tertiary qualities has been defined by philosopher Roberta De Monticelli as “perception or affective sensitivity” because it is the experience that, based on the encounter between the phenomenal structure of the object and the embodied perceptual processes of the subject, allows us to identify those properties of things that can affect us, positively or negatively. We have direct access to the perceptive units of objects endowed with certain values or information for us, independently of previous experiences, inferences, or cultural transmission. Thus, we will say that we “see” in a lush tree a place to shelter from the heat of the sun’s rays, and in the gentle curves of hills dotted with vegetation and adorned with a watercourse a place where we can be, pleasantly and comfortably.

As highlighted by Sinico (2020), also referring to Köhler (1938) in relation to landscape perception, tertiary qualities are also “expressive” qualities because the landscape expresses and externalizes its essential character through these perceptible properties. So, it is not about meanings projected by the perceiver, but about characteristics inherent to the object that present themselves with objectivity in the experience, and which are also found in the methodology of inter-observation used by experimental phenomenology (Bozzi 1978, translated in Bianchi and Davies 2019, pp. 198–210; Kubovy 2003, pp. 579–86).

At this point, it is impossible not to think of the more recent contribution of James J. Gibson (1979), who, as a student of Koffka, introduced, in his ecological theory of perception, the idea that perception is the result of “ecological” characteristics of the environment, namely the molar structures of the environment that function as informative capacities, which the environment possesses and which necessarily emerge in the perception process by the perceiving subject, as environmental perception is organized in such a way that the characteristics of the environment reach the subject as “understandable.” Gibson condenses this view into the concept of “affordances,” which are characteristics of stimuli that “tell us what we can do with them” (Gibson 1979, p. 138) and serve as a guide to the subject’s perception, which—it should be noted—is perception aimed at action and interaction with these objects. Affordances are, therefore, qualities of objects that represent the opportunities and obstacles that objects can offer to our actions. Grasping these qualities means, as De Monticelli and Conni (2008) says, being affected by them, more or less pleasantly or unpleasantly.

Thus, a certain perceptive characteristic corresponds to a certain expressive characteristic. It is indisputable that there are environmental characteristics that, in interaction with the human perceptual/affective system—which, as seen before, is embodied—can render the environment restorative. According to Paolo Bozzi’s vision of tertiary qualities, we can assume that some environments, because of their own perceptual characteristics, express some kind of a message that sounds like “Here you can feel good, relax, regenerate, satisfy your needs”.

As illustrated by Sinico (2015), communication can be mediated by signs or perception, based on the expressive qualities of the object. From this perspective, the natural environment, pre-existing human action, may represent a clear example of perceptual communication not mediated by mental representations (Sinico 2019). Therefore, what we grasp, the affectively connoted meanings that “come to us” when encountering the environment, can only be rooted in the environment’s own phenomenal properties, thus in its perceptual characteristics, or in configurations of characteristics and the relationships between them, organized in perceptual gestalts. As a result, the restorative capacity of the natural environment can only stem from the encounter between the phenomenal structures of the environment itself and our embodied perceptual and affective system.

While it is an unexplored domain to determine which perceptual characteristics of the environment underlie the expressive quality we define as restorative, it is worth highlighting that previous research (e.g., Wolfe 1994, 1996; Wolfe et al. 2007; Wolfe and Horowitz 2017) has examined the elements of the phenomenal scene that automatically attract attention and those that require deliberate exploration. Additionally, other studies (e.g., Oliva and Torralba 2001; Greene and Oliva 2009) have proposed a model for recognizing phenomenological scenes that bypasses the need for segmenting and processing individual objects or regions. Such a perspective is consistent with the reasoning that the restorative effect held by certain environments is related to their tertiary qualities, and raises the question of what specific characteristics, relationships among expressive features, or even gestalts of expressive environmental attributes lead to restorative effects. Therefore, the topic of the tertiary qualities characterizing restorative environments should be investigated in future research in the field of restorativeness.

Some data have been collected to answer this question, but certainly more evidence is needed, as well as a widening of the question to include different kinds of environments in the inquiry about the tertiary qualities that demonstrate restorative effects. For instance, the meta-analysis by Menardo et al. (2021) reveals that natural environments consistently result in higher restorativeness compared to urban environments, regardless of the characteristics of the observer, the instrument used to measure restorativeness, the mode of experience (real or simulated environment), and even the presence of human-made alterations (the results show that natural environments within an urban setting still maintain their restorative capacity). However, different environmental characteristics, such as the presence of vegetation, water bodies, and the quantity and type of light, correspond to different levels of restorativeness, suggesting that the same authors indicate the exploration of environmental variables influencing the perception of restorativeness as a direction for future research.

## 5. Bridging the Gap: Integrating Explanatory Theories of Restorativeness with the Phenomenological and the Embodied Cognition Perspectives

In the preceding paragraphs, we explored theories that offer possible explanations for the phenomenon of the restorative capacity of the natural environment (SRT and ART), and then delved into the perception of the individual–environment relationship based on the embodied paradigm, showing that the basis of perceptual and affective processes is not a cognitive substrate but rather the experience of interaction with the environment itself. Perceptual processes are thus simulated incarnations of experience and, as such, essentially have an affective nature because they are affectively connoted—as the affective dimension represents the basic guidance system for orienting our action in the world—in all our experiences of reality.

At this point, we wish to propose an integration of the explanatory theories of restorativeness, and a phenomenological view of it, with the contribution of the embodied vision of perceptual and individual–environment interaction processes.

First, let us review the two traditional theories of SRT and ART in light of this attempt at integration.

The “psycho-evolutionary” framework proposed by Ulrich (1983) and Ulrich et al. (1991) highlights that the restorative power of the natural environment is based on an immediate positive affective response (aesthetic preference). However, in addition to aesthetic preference, it also involves an “immediate, unconsciously triggered and initiated” emotional response that affects physiological arousal levels, attentional and conscious processing, and behavior. Both preference and emotional response have an affective nature and would be triggered by a specific arrangement of environmental properties that “suggest” to the perceiving subject that the environment possesses favorable characteristics for survival and well-being. In phenomenological terms, the immediate and non-cognitively mediated stress reduction response would be triggered by the phenomenal properties of the environment (specifically, the presence of vegetation and water elements) that communicate to the perceiver, “I can meet your essential needs for protection and nourishment,” and “suggest” a favorable interaction with the environment for the individual.

Looking at Kaplan and Kaplan’s (1989) theory of environmental preference, we notice that the four factors they identified as the basis of environmental preference are phenomenal qualities of the environment itself that trigger immediate affective responses and suggest possible modes of interaction compatible with the individual’s needs. According to Kaplan’s view, a preferred environment has four characteristics: coherence, legibility, complexity, and mystery, meaning that it “immediately appears” as controllable, supportive, and restorative. In her subsequent work “The restorative benefits of nature: toward an integrative framework,” published in 1995, Kaplan lays the foundations for the restorativeness in fascination. That is, she identifies a possible explanation for the restorative effect of the natural environment in the characteristics of the environment that allow us to shift from a mode where direct attention is used to a mode where involuntary attention is used, which requires no effort and therefore restores attentional processes. The other three characteristics of the environment that influence the individual–environment relationship towards generating a restorative effect are: (1) being away; a new or different environment or even the old environment viewed in a new way, (2) extent; the environment must be rich and coherent enough to constitute a whole other world, and (3) compatibility or being responsive; there should be compatibility between the environment and one’s purposes and inclinations. As seen, these are all tertiary phenomenal properties. Furthermore, Kaplan emphasizes that “there is overwhelming evidence that information processing can occur rapidly and without consciousness” (p. 177), highlighting that the perception of these characteristics of the environment is not the result of cognitive processing but emerges directly from the perceptual process, presenting itself to the perceiver immediately. Finally, while Kaplan hopes for a reconciliation and integration between the Stress Reduction Theory and the Attention Restoration Theory, she still emphasizes that experiences of stress (central to SRT) and fatigue (central to ART) have profound “phenomenological differences” (p. 180).

Regarding the proposed reconciliation between the two theories, Joye et al.’s (2016) contribution defines stress as an experience of the “perceptual inadequacy” of the environment in relation to the individual’s resources. Therefore, they propose overcoming the divergence between ART and SRT with the theory of the Perceptual Fluency Account (Joye and van den Berg 2018). The central assumption of the PFA is that natural environments are processed more fluently than urban or human-made environments, and this fluency results in a differential restorative potential. Thus, the natural environment would possess information redundancy that makes its visual processing more fluent, thereby favoring the perceptual process. Therefore, a restorative environment would be one that possesses certain characteristics that “feel better” to us, both for evolutionary reasons and due to perceptual processing (Joye and van den Berg 2011). The Perceptual Fluency Account, therefore, considers both stress reduction and the restoration of attention as secondary effects of perceptual fluency. Consequently, as indicated by the authors, “one of the main challenges of PFA is to pinpoint exactly which (visual) features make natural scenes more fluent than urban scenes” (p. 267).

Finally, let us mention two more theories that have proposed an explanation for the restorative capacity of the natural environment, included here for their apparent connection to an embodied view of the individual–environment relationship. The construct of “Connectedness to Nature” allows us to overcome the dichotomy between ART and SRT by arguing that individuals can experience a sense of well-being specifically through the development of a sense of purpose and identity, feeling a connection to nature and recognizing themselves as part of it. The “Micro-Restorative Experiences and Instorative Effects” approach also overcomes both SRT and ART by suggesting that when individuals have a perceptual contact with the natural environment, they can create a repository of experiences that contribute to combating stress and can even be “instorative,”, i.e., regenerating, revitalizing, and invigorating. This effect is observed even in short-duration contacts, through visual means (real or mediated—think of the enjoyment of photographs or video stimuli, as well as virtual reality stimuli) and also through other sensory modalities (auditory or olfactory).

Another notable contribution is Rathunde’s (2009), which proposes another potential model of explanation for the restorative capacity of nature, rooted in the embodied paradigm but integrating elements attributable to a phenomenological approach. A view of the embodied mind entails that the process of constructing meaning from reality begins in the most primitive sensory–motor processes. Therefore, the foundation of knowledge has a profoundly and originally affective and aesthetic nature, in terms of primary responses of approach/avoidance. The emotional experience represents a primary, pre-cognitive response to the environment (Zajonc 1980). We first experience how a situation “makes us feel,” and this response originates from encountering the pervasive qualities of the stimulus’ situation. According to Johnson (2007), “If you pay attention to how your world shows itself, you will indeed see the flow of experience comes to us as unified wholes (gestalt) that are pervaded by an all-encompassing quality that makes the present situation what and how it is” (p. 73). This has been called “physiognomic perception” (Werner 1956), a mode of immediate perception based on embodied sensory–motor processes, allowing the perceiver to emotionally connect with the situation being experienced, eliciting an affective response. Within this framework, the ability of natural environments to restore attention comes from the fact that nature helps integrate the processing system. By engaging the part of the system ontologically preceding selective attention and abstract processing, the part connected to affective responses, the aesthetic perception of the pervasive qualities of the situation allows for the recovery of the quality and strength of selective attention and concentration processes, thereby producing a restorative effect.

## 6. Conclusions

The present contribution aims to consider the topic of restorativeness from an integrated and integrative perspective. Specifically, it intended to look at the restorative effects of the human–environment relationship, typically explained from a psychoevolutionary perspective (Ulrich 1983; Kaplan 1995), in light of, on the one hand, the approach of experimental phenomenology, and, on the other hand, the framework of embodied and enactive theories.

The effort to bring together these three views has driven us to look at the phenomenic qualities of the environment as expressive qualities, and at perceptual processes as a way to come to know the environment that are immediately imbued with affective and aesthetic connotations. All this is with an awareness that the relationship between humans and the natural environment, more than any other human–environment relationship, cannot be considered separately from an evolutionary perspective, which places and reads such a relationship within the evolutionary path of the human species.

Our proposal is that the integration of the presented views brings us back to some fundamental points, which, to our knowledge, have not been considered so far when addressing the question of the restorative power of the natural environment, with its positive effects on individual well-being.

The first point concerns the fact that, when considering the restorative power of certain environments, the interdependence between the individual and the environment is of primary importance. The laws of Gestalt psychology, which have explained the functioning of perception as focusing on the relationship between stimulus and context (Wertheimer 1912), insightfully suggest looking at the individual and their environment as a unit, and such an idea should be placed at the center of reflection on the restorative power of natural environments. Embodied and enactive theories emphasize the unity between individual and environment when describing, on the one hand, perception as intimately connected to action in the environment and on the environment, and, on the other hand, defining the mind as building itself in relation to the environment (“extended mind”, Clark and Chalmers 1998). Therefore, perception qualifies as an intrinsically relational process. There is no perception without an environment to relate to; hence, our proposal is that the restorative effect of the environment can be understood in light of the fact that perception of that environment is perception of the relationship with it (Gallese 2005).

A second point is that the new frontier of research on restorativeness is to investigate the characteristics of the environment, or rather the set of the environmental elements and the relationships that connect them (“field dynamics”, Köhler 1929) that, when entering into relationship with the individual, produces a restorative effect.

The last point, closely related to the previous one, is that in the developments of research on restorativeness, it is necessary, as Mungan (2023) puts it, to address “the challenge of including the first-person experience as an essential part of understanding the cognizing being” (p. 13). To achieve this aim, we assume that inter-observation, typical of experimental phenomenology, can be considered the elective method. Already successfully applied to the study of other processes, such as perception, problem-solving, and creativity, if used in the study of the restorative power of the natural environment, interobservation (Bozzi 2002) will allow for the collection of valuable data on the subjective experience of restorativeness. Interestingly, restorativeness has been studied based on paradigms that guided the collection of both behavioral and psychophysiological data. However, as the phenomenal experience of the environment is primarily affective, in agreement with both the embodied perspective (Varela et al. 2017) and the perspective of experimental phenomenology (Bozzi 1989), our view is that the relationship between individual and environment needs to be understood by starting with an examination of the dynamics underlying the subjective experience of a restorative environment.

## Data Availability

Not applicable.
